# Sporadic neurofibroma of facial nerve presenting as parotid gland tumor: a rare case report

**DOI:** 10.1093/jscr/rjae434

**Published:** 2024-07-02

**Authors:** Shko H Hassan, Karzan M Salih, Abdulwahid M Salih, Aras J Qaradakhy, Ari M Abdullah, Yadgar A Saeed, Aso S Muhialdeen, Imad J Habibullah, Hardi M Dhahir, Fahmi H Kakamad, Masty K Ahmed

**Affiliations:** Scientific Department, Smart Health Tower, Madam Mitterrand Street, Sulaymaniyah, Kurdistan 46001, Iraq; Scientific Department, Smart Health Tower, Madam Mitterrand Street, Sulaymaniyah, Kurdistan 46001, Iraq; Scientific Department, Smart Health Tower, Madam Mitterrand Street, Sulaymaniyah, Kurdistan 46001, Iraq; Department of Surgery, College of Medicine, University of Sulaimani, Zanko Street, Sulaymaniyah, Kurdistan 46001, Iraq; Scientific Department, Smart Health Tower, Madam Mitterrand Street, Sulaymaniyah, Kurdistan 46001, Iraq; Department of Radiology, Shorsh Teaching Hospital, Shorsh Street, Sulaymaniyah, Kurdistan 46001, Iraq; Scientific Department, Smart Health Tower, Madam Mitterrand Street, Sulaymaniyah, Kurdistan 46001, Iraq; Department of Pathology, Sulaymaniyah Teaching Hospital, Zanko Street, Sulaymaniyah, Kurdistan 46001, Iraq; Scientific Department, Smart Health Tower, Madam Mitterrand Street, Sulaymaniyah, Kurdistan 46001, Iraq; Scientific Department, Smart Health Tower, Madam Mitterrand Street, Sulaymaniyah, Kurdistan 46001, Iraq; Scientific Department, Smart Health Tower, Madam Mitterrand Street, Sulaymaniyah, Kurdistan 46001, Iraq; Scientific Department, Smart Health Tower, Madam Mitterrand Street, Sulaymaniyah, Kurdistan 46001, Iraq; Scientific Department, Smart Health Tower, Madam Mitterrand Street, Sulaymaniyah, Kurdistan 46001, Iraq; Department of Surgery, College of Medicine, University of Sulaimani, Zanko Street, Sulaymaniyah, Kurdistan 46001, Iraq; Kscien Organization, Hamdi Street, Azadi Mall, Sulaymaniyah, Kurdistan 46001, Iraq; Scientific Department, Smart Health Tower, Madam Mitterrand Street, Sulaymaniyah, Kurdistan 46001, Iraq

**Keywords:** intraparotid gland neurofibroma, parotid gland, NF1, facial nerve, neurofibromatosis, parotid tumor

## Abstract

Intraparotid gland neurofibroma is a rare benign tumor that arises from Schwann cells of the facial nerve within the parotid gland. This case report discusses a 41-year-old woman who experienced a painless preauricular swelling on her right side for over 5 years. Clinical examination and ultrasound revealed a well-defined mass in the parotid gland. The patient underwent total mass excision, resulting in transient facial nerve dysfunction but complete recovery. These tumors often manifest as solitary masses in the parotid region and may compress nearby structures, causing facial paralysis or numbness. Their diagnosis can be challenging due to similarities with other parotid gland tumors and possible associations with neurofibromatosis. Managing intraparotid tumors, including neurofibromas, involves a multidisciplinary approach with input from cytopathologists, radiologists, and surgeons.

## Introduction

Intraparotid gland neurofibroma (IGN) is a rare benign tumor that originates from the Schwann cells of the facial nerve within the parotid gland, although it differs from schwannoma in that it contains not only neoplastic Schwann cells but also additional non-neoplastic cells, like mast cells, perineural-like cells, residual axons, and fibroblasts. The exact cause of IGN is not well understood, but it is believed to be either sporadic or associated with neurofibromatosis (NF) [1]. NF is a genetic disorder characterized by the growth of tumors on the nerves, and it may present with IGN or schwannomas [2].

In terms of common epidemiology, the frequency of parotid tumors originating in the facial nerve, including IGN, has been estimated to be between 0.2% and 1.55% [3, 4]. IGN mainly affects adults, with a peak incidence occurring in the fourth to sixth decades of life. In rare instances, IGN may also occur in children and adolescents. However, the occurrence of this condition is relatively uncommon among pediatric patients compared to adults [5]. Typically, patients present with an asymptomatic parotid gland mass that grows slowly over time. There are no specific features that differentiate it from other common benign tumors of the parotid gland, like pleomorphic adenoma. Histopathological examination gives the definitive diagnosis [1].

The current case report aims to present a rare occurrence of IGN in a middle-aged patient and provide a comprehensive review and analysis of its clinical presentation, diagnosis, and management.

## Case presentation

A 41-year-old female presented with a painless right-sided preauricular swelling for the last 5 years. The size of the swelling increased over time. Prior medical and surgical histories were insignificant. On inspection, there was a swelling that involved the right preauricular region on palpation. The swelling was firm without tenderness and had normal function in all branches of the facial nerves. There was no numbness or paresthesia. General physical examination was normal without any evidence of skin lesions. Blood investigations in the form of CBC, liver function tests, lipid profiles, blood glucose, and thyroid function tests were normal. Neck ultrasound (US) revealed a modestly enlarged right parotid gland with normal parenchymal echogenicity and vascularity, as well as a well-defined, thin lobulated surface, hypoechoic, and hypovascular mass of 35 mm involving the superficial and deep lobes, with the normal surrounding tissue. The mass was situated between the superficial and deep layers of the parotid gland, overlaying the main trunk of the facial nerve. However, it is important to note that while US was effective for visualizing the facial nerve canal within the temporal bone and surrounding structures, it could not directly visualize the facial nerve itself.

**Figure 1:**
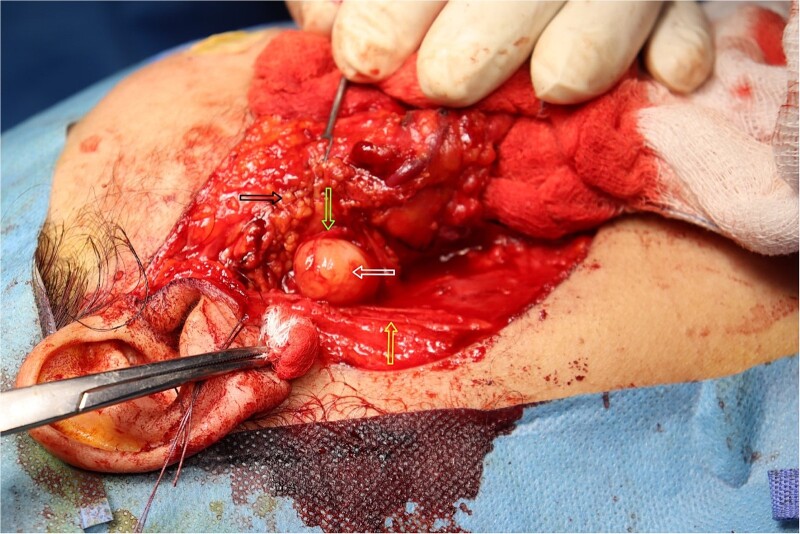
Intra-operative picture of the mass over the main trunk of the facial nerve. The white arrow indicates the mass over the main trunk of the facial nerve. The yellow arrow points to the great auricular nerve. The green arrow highlights a branch of the facial nerve. The black arrow shows the parotid gland.

**Figure 2:**
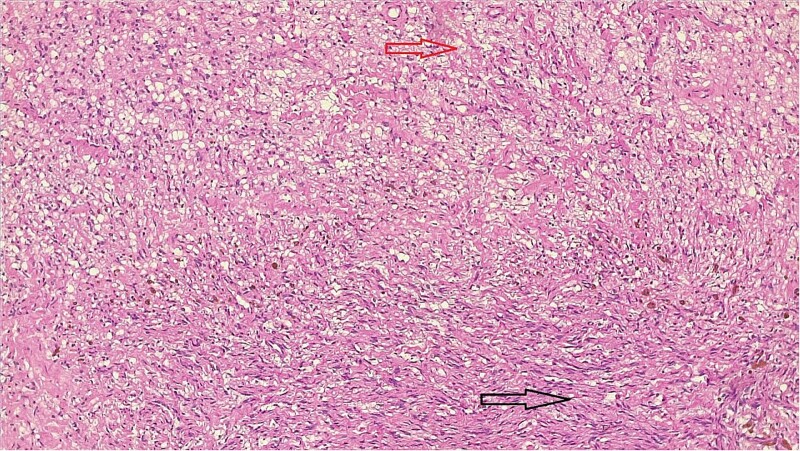
Bland-looking cellular spindle cells (dark arrow) infiltrating into adjacent fibrofatty tissue (yellow arrow), Hematoxylin, and Eosin stain. Microscopic power 4 × 10.

**Figure 3:**
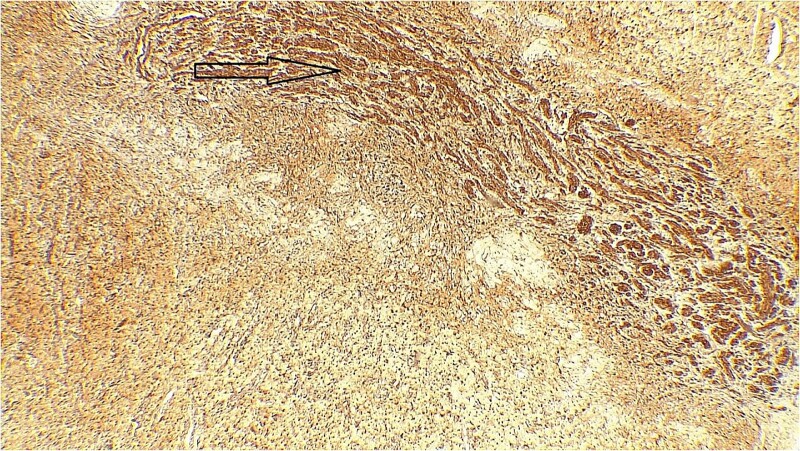
Immune stain S100, strong nuclear and cytoplasmic staining pattern in the tumor cells.

The result of the fine needle aspiration (FNAC) was a bland-looking spindle cell lesion, suggestive of a neural origin lesion like schwannoma. The patient was positioned in a supine position after she received general anesthesia. The face was turned to the opposite side of the tumor with a slight extension of the neck. An S-shaped incision was carried out in the front of the ear to the ear lobule, then it curved around the mastoid process posteriorly near the mastoid process and swung to the upper cervical creases smoothly. The cervical-facial skin flap was elevated with dissection to expose the tumor adequately, and then a traction suture was placed over the earlobe to widen the visual field for the operator. Dissection was carried out until the tumor was completely exposed, showing a smooth, ovoid, and whitish mass along the main trunk of the facial nerve (Fig. 1). The nerve was seen entering and exiting the posterior aspect of the mass on both sides. The layers over the mass were opened, and the mass was enucleated, leaving a flattened nerve posteriorly. No parotid gland excision was carried out. The postoperative histopathological examination revealed neurofibroma (Fig. 2) with a positive expression for S100 immune stain (Fig. 3). Postoperatively, the patient developed moderate (grade III) facial nerve dysfunction with obvious but not disfiguring deformity of the right side of the face, likely due to the pressure effect of the mass or traction during the operation. Two months after the operation, the facial nerve status improved to grade one, and after 4 months, she developed full recovery of facial nerve function.

## Discussion

Salivary gland neoplasms may also affect the branches of the facial nerve. Usually, there are two types of these benign tumors: neurofibroma, which is uncommon, and schwannoma, which is the more frequent one. The majority of them come from Schwann cells, resulting in a condition called IGN [6]. The occurrence of IGN can be observed in different parts of the seventh cranial nerve, ranging from the cerebellopontine angle to its terminal branches on the face [7]. While most cases are localized within the intra-temporal segment of the facial nerve, only a small percentage (9%) occurs in extratemporal segments [8, 9]. In general, IGNs are painless masses that appear as solitary tumors in the parotid region. These tumors can be difficult to diagnose since they resemble other types of parotid gland tumors. They typically grow slowly and may not initially cause any symptoms. Also, its presentation depends on the size and location of the tumor. As the tumor expands, it can compress surrounding structures and lead to symptoms, such as facial weakness or numbness, earache on the same side of the tumor, and, in severe cases, hearing loss or tinnitus. Usually, this condition is associated with NF type 1 (NF-1), a common inherited genetic disorder with an autosomal-dominant inheritance form. The gene responsible for NF-1 is on chromosome 17. Multiple neural tumors, Lisch nodules, and “cafe´ au lait” are the major characteristic features of this genetic disorder [3, 10, 11]. The current case was a middle-aged female who presented with a painless swelling in the right parotid region for more than 5 years. She did not report any associated symptoms, such as facial weakness, numbness, hearing problems, or any specific lesions elsewhere in her body.

FNAC is commonly used as an initial diagnostic tool for IGN [12]. The results from FNAC can provide valuable information regarding the cytological features of the tumor, helping to differentiate it from other parotid gland tumors [13]. Additionally, FNAC can help assess the cellular composition of the tumor, such as the presence of spindle cells characteristic of neurofibromas, as well as the presence of any atypical or malignant cells [14]. Furthermore, immunohistochemical staining can be performed on the obtained samples to confirm the diagnosis and distinguish it from other intra-parotid tumors [15]. However, it is important to note that FNAC may not always provide a definitive diagnosis, and histopathological examination of the excised tumor is often required for confirmation [16]. In the present study, FNAC was performed and suspected a neural origin lesion like Schwannoma.

The radiological appearance of IGN can vary depending on the imaging modality used, such as US or magnetic resonance imaging (MRI) [17]. For US, IGN typically appears as hypoechoic masses with irregular borders [11]. They may also demonstrate increased vascularity on Doppler imaging. Additionally, US can help in assessing the size and extent of the tumor within the parotid gland, as well as any associated changes to the surrounding structures [18]. On MRI, they often demonstrate low-to-intermediate signal intensity on T1-weighted images and high signal intensity on T2-weighted images [18]. In the current case, a neck US revealed a well-defined, thin well-lobulated surface, hypoechoic, and hypovascular mass of 35 mm involving the superficial and deep lobes, with normal surrounding tissue. Preoperative MRI was not done.

Surgical management is the mainstay treatment for IGN [19]. This typically involves a partial or total parotidectomy, with careful dissection to preserve the facial nerves and minimize the risk of postoperative complications. In some cases, due to the benign nature of these tumors, a conservative approach may be taken, and regular monitoring is recommended [10]. However, if the tumor causes significant symptoms or there are concerns about malignancy, surgical excision is usually recommended [20]. Furthermore, according to genuine literature, recent advancements in surgical techniques have allowed for more precise and minimally invasive approaches to the resection of these tumors, leading to improved outcomes and facial nerve preservation [21, 22]. Additionally, the use of intraoperative neuromonitoring can play a crucial role in ensuring the preservation of the facial nerve during surgery [23]. Moreover, postoperative follow-up and monitoring are crucial for detecting any signs of recurrence or complications. Immunohistochemistry for S100 staining yields strongly positive results for IGN [10, 24]. In the current case, a total excision of the tumor was done, and the postoperative histopathological examination showed NF with a positive result of the S100 immune stain.

In conclusion, the rarity of IGN mimicking other parotid gland tumors emphasizes the significance of a comprehensive diagnostic approach.
